# Targeted deep sequencing from multiple sources demonstrates increased NOTCH1 alterations in lung cancer patient plasma

**DOI:** 10.1002/cam4.2458

**Published:** 2019-08-01

**Authors:** Yuwei Liao, Zhaokui Ma, Yu Zhang, Dan Li, Dekang Lv, Zhisheng Chen, Peiying Li, Aisha AI‐Dherasi, Feng Zheng, Jichao Tian, Kun Zou, Yue Wang, Dongxia Wang, Miguel Cordova, Huan Zhou, Xiuhua Li, Dan Liu, Ruofei Yu, Qingzheng Zhang, Xiaolong Zhang, Jian Zhang, Xuehong Zhang, Xia Zhang, Yulong Li, Yanyan Shao, Luyao Song, Ruimei Liu, Yichen Wang, Sufiyan Sufiyan, Quentin Liu, Gareth I. Owen, Zhiguang Li, Jun Chen

**Affiliations:** ^1^ The Second Hospital of Dalian Medical University Dalian China; ^2^ Center of Genome and Personalized Medicine, Institute of Cancer Stem Cell Dalian Medical University Dalian China; ^3^ Advanced Institute for Medical Sciences Dalian Medical University Dalian China; ^4^ The First Affiliated Hospital of Dalian Medical University Dalian China; ^5^ Faculty of Biological Sciences Pontificia Universidad Católica de Chile Santiago Chile; ^6^ The Second Affiliated Hospital, School of Medicine Zhengzhou University Zhengzhou China

**Keywords:** lung cancer, next‐generation sequencing, NOTCH1, plasma, pleural effusion

## Abstract

**Introduction:**

Targeted therapies are based on specific gene alterations. Various specimen types have been used to determine gene alterations, however, no systemic comparisons have yet been made. Herein, we assessed alterations in selected cancer‐associated genes across varying sample sites in lung cancer patients.

**Materials and Methods:**

Targeted deep sequencing for 48 tumor‐related genes was applied to 153 samples from 55 lung cancer patients obtained from six sources: Formalin‐fixed paraffin‐embedded (FFPE) tumor tissues, pleural effusion supernatant (PES) and pleural effusion cell sediments (PEC), white blood cells (WBCs), oral epithelial cells (OECs), and plasma.

**Results:**

Mutations were detected in 96% (53/55) of the patients and in 83% (40/48) of the selected genes. Each sample type exhibited a characteristic mutational pattern. As anticipated, TP53 was the most affected sequence (54.5% patients), however this was followed by NOTCH1 (36%, across all sample types). EGFR was altered in patient samples at a frequency of 32.7% and KRAS 10.9%. This high EGFR/ low KRAS frequency is in accordance with other TCGA cohorts of Asian origin but differs from the Caucasian population where KRAS is the more dominant mutation. Additionally, 66% (31/47) of PEC samples had copy number variants (CNVs) in at least one gene. Unlike the concurrent loss and gain in most genes, herein NOTCH1 loss was identified in 21% patients, with no gain observed. Based on the relative prevalence of mutations and CNVs, we divided lung cancer patients into SNV‐dominated, CNV‐dominated, and codominated groups.

**Conclusions:**

Our results confirm previous reports that EGFR mutations are more prevalent than KRAS in Chinese lung cancer patients. NOTCH1 gene alterations are more common than previously reported and reveals a role of NOTCH1 modifications in tumor metastasis. Furthermore, genetic material from malignant pleural effusion cell sediments may be a noninvasive manner to identify CNV and participate in treatment decisions.

## Graphical abstract

Targeted deep sequencing for 48 tumor‐related genes was applied to 153 samples from 55 lung cancer patients obtained from six sources. NOTCH1 exhibited mutational changes in 70 % plasmas, much higher than in tumor tissue (20%), highlighting the extraordinary usefulness of NOTCH1 in liquid biopsy.

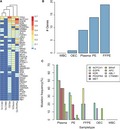

## 1 INTRODUCTION

Lung cancer is one of the commonly diagnosed cancers.^1^, ^2^ The principal types of lung cancer are non‐small cell lung cancer (NSCLC) and small cell lung cancer (SCLC),^3^, ^4^ with a combined expected incidence of 234 030 and mortality of 154 050 in the United States in 2018.^5^ Recurrent malignant pleural effusion (PE) is associated with worsening quality of life in lung cancer patients; the overall survival time in patients with PE is estimated to be only 4.3 months.^6^ Patients with PE usually report breathlessness, chest pain and a cough which often make thoracentesis or chest tube drainage necessary. As a result, a PE biopsy is less invasive and can thus be obtained more frequently. But only traditional techniques such as fluorescent in situ hybridization, PCR or amplification refractory mutation system, and pyrosequencing have been applied in analyzing pleural effusion supernatant (PES) and pleural effusion cell (PEC) samples.^7^, ^8^, ^9^, ^10^, ^11^, ^12^ New techniques with high throughput and sensitivity need to be employed to evaluate the clinical value of pleural effusion.

Most of the lung cancer genome studies have been performed on samples of tumor tissue (FFPE or fresh frozen tissue).^13^, ^14^, ^15^, ^16^ These studies did not deliver the overall mutation patterns due to the presence of intra‐ and inter‐tumor heterogeneities.^17^, ^18^ In contrast, PE can overcome such limitations and reveal the combined mutation pattern representative of different tumors in the lung.^19^ Plasma, due to its whole‐body circulation, carries mutations from even more diverse sites, such as distant metastasis.^20^

In addition to single nucleotide variants (SNV), copy number variation is one of the major chromosome aberrations of the cancer genome. The noninvasive extraction of blood has previously permitted the evaluation of SNVs in cfDNA.^20^, ^21^, ^22^ However, it is challenging to analyze CNVs from cfDNA samples, as this source is usually highly fragmented^23^ and this fragmentation is associated with cell apoptosis and nucleosome occupancy.^24^ The read depth in sequencing analysis may not only reflect copy number alterations in tumor cells but also the nucleosome occupancy (a given region of DNA that is occupied by a histone octamer). The samples that could be non‐invasively obtained and detect CNVs would greatly benefit precision medicine.

Significant differences are evident in the prevalence of mutations in patients of different ancestries. In a comprehensive characterization of the somatic mutations in lung adenocarcinoma from 230 Caucasian patients,^16^ TP53 was mutated with the highest frequency (46% of patients), followed by KRAS (33%) and EGFR (14%). However, in a cohort of lung cancer patients of Chinese ethnicity, the most frequently mutated gene was EGFR (46.7% of patients), followed by TP53 (21.2%), ALK (12.1%) and KRAS (10.1%).^25^ These studies were based on tumor tissues. It is currently unknown if such ethnicity‐related differences are maintained in liquid biopsy samples.

In this study, we compared somatic mutations from six sources of samples including PES, PEC, formalin‐fixed paraffin‐embedded (FFPE) tumor tissues, white blood cells (WBCs), oral epithelial cells (OECs), and plasma, using deep sequencing of targeted genome composed of the full length of 48 cancer‐related genes. Copy number variants were also identified in the PEC samples. This study demonstrated the unique values of each sample type for personalized medicine of lung cancer, and will benefit the clinical implementation of liquid biopsies.

## 2 MATERIAL AND METHODS

### 2.1 Patient cohort

The study was approved by the Second Affiliated Hospital of Dalian Medical University and the protocol conformed to the ethical guidelines of the 1975 Declaration of Helsinki. Written informed consent was obtained from each patient. Samples were collected from 55 Chinese lung cancer patients at Dalian, China. All patients were diagnosed at stage IV and resented thoracic metastases. In total, PES was sampled from 40 patients, PEC from 47 patients, plasma from 10 patients. FFPE tumor tissues, peripheral blood and OECs were also sampled when possible (Figure S1A, Tables S1 and S2).

### 2.2 DNA extraction

The PE and plasma samples were centrifuged, and cell‐free supernatant (PES) and cell pellets (PEC) were collected, respectively. Genomic DNA was extracted from OEC, PEC and WBC using TIANamp Genomic DNA Kit according to the manufacturer's protocols (TIANGEN). Cell free DNA was extracted from PES and plasma using QIAamp Circulating Nucleic Acid Kit (QIAGEN). Genomic DNA was extracted from the paraffin‐embedded tumor tissue samples using the QIAamp DNA FFPE Tissue Kit (QIAGEN) according to the manufacturer's protocols.

### 2.3 Library preparation

Exonic regions of 48 tumor‐related genes were amplified using multiplex PCR (Table S3), which was performed using 20 ng cfDNA on Veriti 96‐Well Thermal Cycler (Applied Biosystems). Reaction was incubated initially at 98°C for 30 seconds for all samples except WBC sample which took three‐minutes incubation. Fifteen cycles of PCR were performed, each cycle was performed at 98°C and 62°C for 10 seconds and 4 minutes respectively. Then the reaction was held at 4°C. For end repair and A‐tailing of DNA fragments, the mixture composed of 37.5 µL DNA, 5 µL Cut smart, 5 µL ATP, 0.5 µL 100 mmol/L dATP, 1 µL T4 PNK Enzyme and 1 µL of 5 units Klenowexo‐DNA Polymerase was incubated for 1 hour at 37°C in thermocycler (GINGKO), then the products were purified using Universal DNA Purification Kit (TIANGEN) and eluted in 27 µL elution buffer. In ligation step, 25 µL A‐tailed DNA was incubated with 1 µL 50 µmol/L multiplexing adapter, 3 µL 10× T4 DNA ligase buffer and 1 µL 400 units/µL NEB T4 DNAligase (M0202L) at 16°C for two hours or overnight. For AMPure XP beads purification, we added 1× volume of AMPureXP beads (30 µL) and incubated it at room temperature for 5 minutes. Then we discarded the supernatant which contained primer dimers. Beads were washed with 200 µL 80% ethanol for 30 seconds at room temperature and the washing was repeated twice. The beads were dried at room temperature for 3 minutes. Then 25 µL Buffer EB was added to the bead tube, pipetted for 10 times, incubated for 2 minutes at room temperature, and stay on magnetic stand at room temperature for about 5 minutes. Then, 22 µL of supernatant was transferred to a new PCR tube. PCR enrichment was conducted by using High‐Fidelity 2× PCR Master Mix. The reaction was incubated initially at 98°C for 3 minutes and followed by fifteen amplification cycles of 98°C for 20 seconds; 65°C for 15 seconds; 72°C for 3 minutes. Then the reaction was held at 4°C.

### 2.4 Mutation calling

All fastq data were trimmed for primer sequence using an in‐house pipeline. Then fastq files were mapped to human genome 38 (hg38) using bwa‐0.7.12.^26^ One hundred and fifty three samples passed quality control. Mutations were identified using Plasma Mutation Detector‐1.6.6^27^ which used WBC as control to correct the base‐position error rate (Figure S3). The program of Plasma Mutations Detector needs two input files, one for mutation hotspots, and the other for single nucleotide polymorphisms. The hotspot file was generated according to COSMIC high frequency mutations and target drug positions (Table S4). The single nucleotide polymorphism file was based on 1000G phase1 high confidence single nucleotide polymorphisms dataset downloaded from ANNOVAR.^28^ Mutations were annotated by ANNOVAR.^28^ We took into account the synonymous mutation when calculating total mutations as synonymous mutations may alter the transcript splicing or gene regulation.^29^ However, only nonsilent mutations were used for comparing mutation frequencies of EGFR and KRAS with other data sets. Several patients were sampled multiple times (Figure S2), however only the first extracted sample was used for comparison with other datasets.

### 2.5 Mutation load analysis

Tumor mutation load was defined as the number of nonsilent mutations divided by target region size expressed in megabase.^30^ When assessing the association of mutations of a specific gene with patient mutation load, we corrected patient mutation load by subtracting the mutations in the gene of interest from overall mutation load. *P* values between the two groups of patients with or without the mutation of the gene of interest were calculated using *t* test, with significance set as a *P *< .05.

### 2.6 CNV calling

Copy numbers were detected in PEC by multi normalization tool ONCONCV‐6.6^31^ using WBC as control. Four types of copy number variants were defined as following: amplification (copy number ≥4), gain (copy number >2), deletion (copy number 0) and loss (copy number between 0 and 2). Co‐occurrence of copy number variants and mutations were determined using Apriori algorithm.^32^

## 3 RESULTS

### 3.1 Targeted deep sequencing to detect the mutational landscape

**Figure 1:**
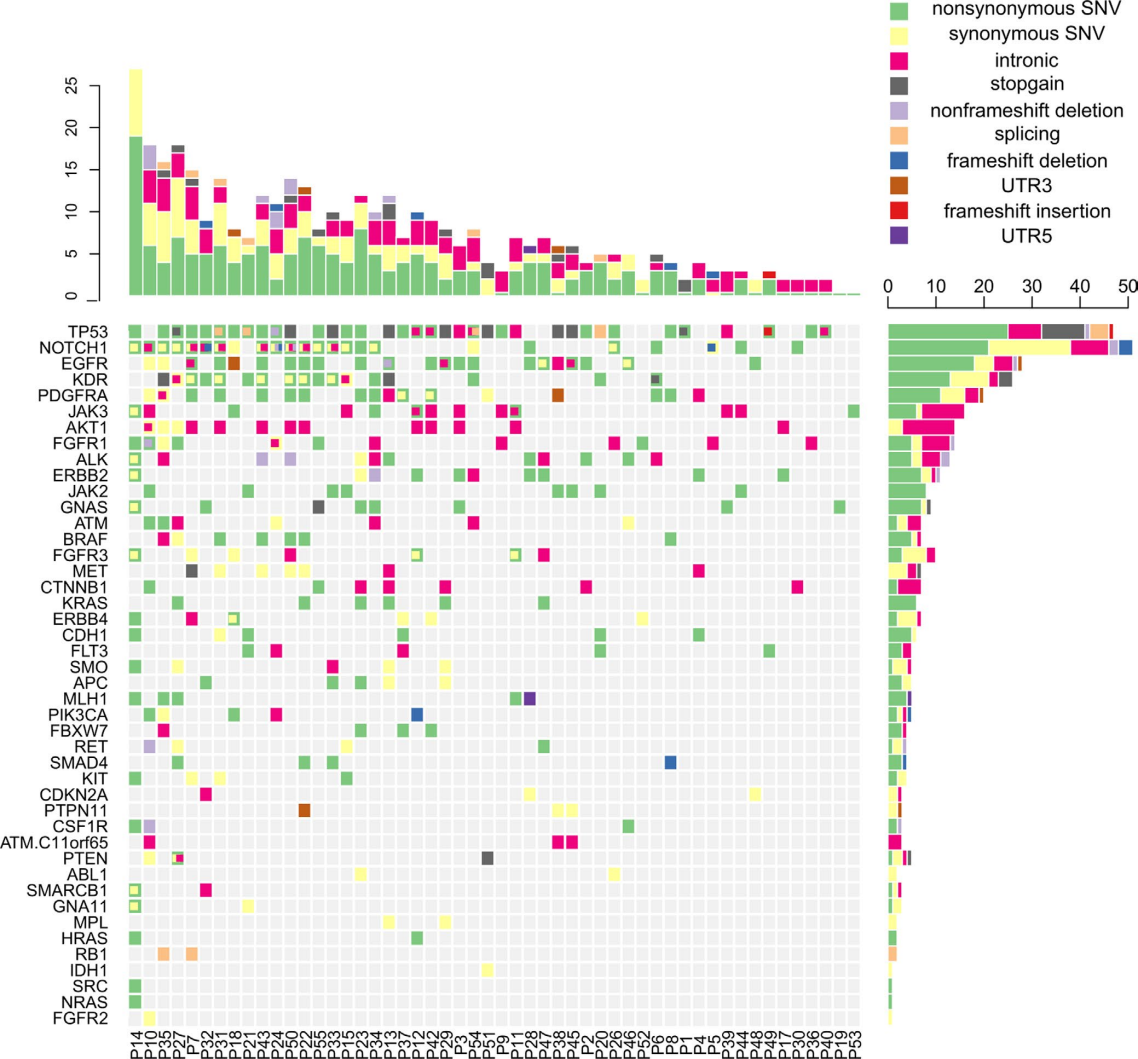
Landscape of lung cancer patient mutations. The column and row represent patients and genes, respectively, and are sorted decreasingly by the number of mutated genes carried by each patient carries (barplot at the top) or the number of patients in which a gene is mutated (barplot at the right). Mutation types are differentiated by colors. Multiple colors are shown in cases where one patient has multiple mutation types in a single gene. Gray denotes no mutations

In total, 153 samples from 55 patients were available for analysis by the 48‐tumor gene panel. Different types of samples were analyzed, including WBC (n = 16), PES (n = 50), PEC (n = 57), OEC (n = 14), plasma (n = 10) and FFPE (n = 6). In many patients samples from differing sources were obtained and some patients had PEC, and PES analyzed at multiple time points during the course of their cancer treatment (Table S1; Figure S1A). Mutation calling was performed using Plasma Mutation Detector, a mutation analysis software with high sensitivity and low error rate.^27^ A total of 595 mutations were detected throughout the samples (Table S5). Mutations were detected in 96% (53/55) of patients and in 83% (40/48) of the genes sequenced. Although nonsynonymous SNV was the most prominent mutation type, some genes exhibited distinct mutational signatures. A high proportion of stop gain mutations was observed in TP53 gene (26% patients) and KDR gene (18%; Figure 1). Notable for the high proportion of synonymous and intronic alterations were AKT1 (100% patients) and NOTCH1 (79%) (Figure 1), indicating that these two genes may participate in the process of tumorigenesis via altering pathways that do not rely on changes in amino acid sequence.

### 3.2 Alteration Frequency, hotspots and mutation load

**Figure 2:**
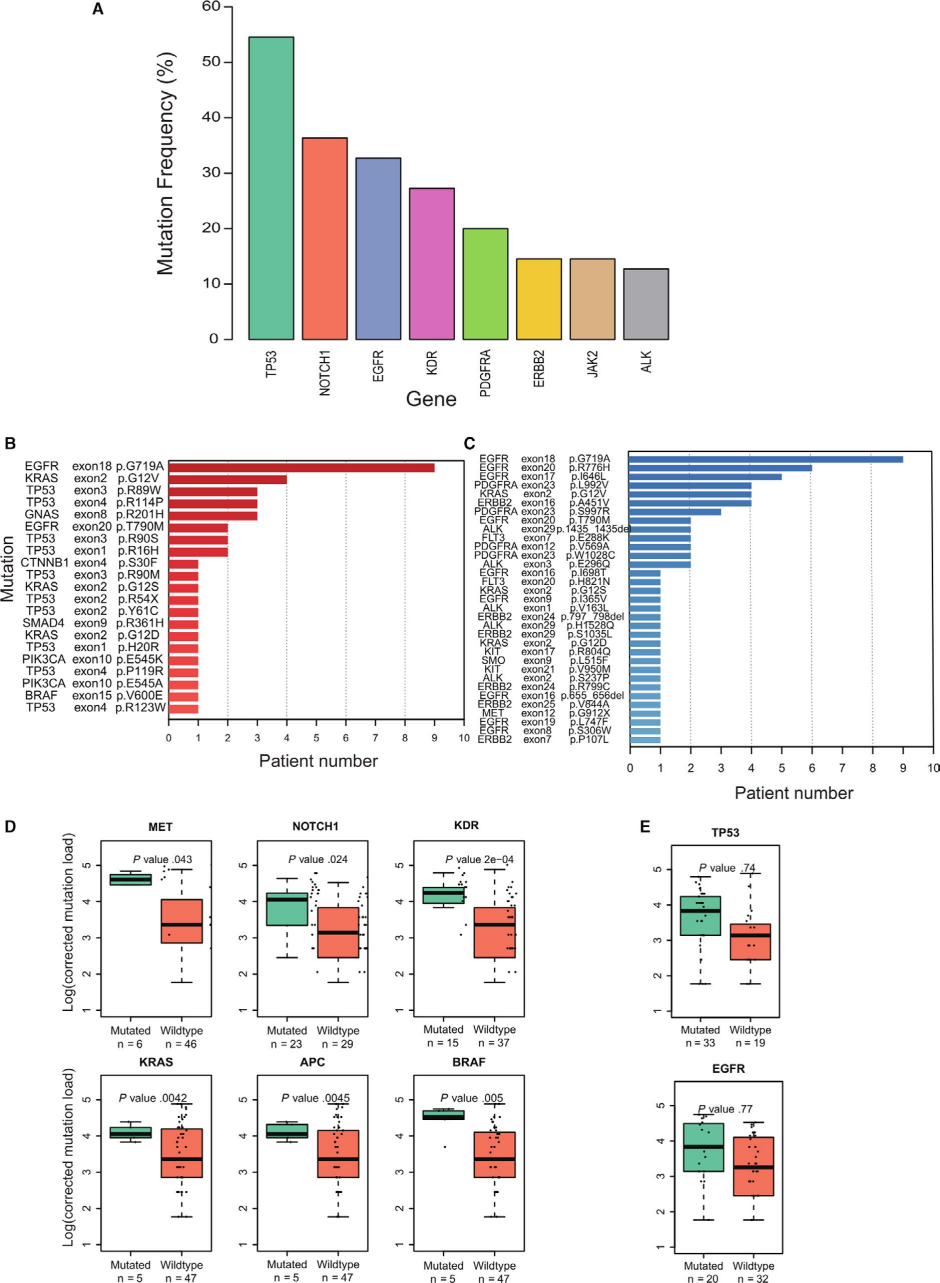
The top eight genes displaying mutational frequency. Mutation frequency refers to the percentage of patients in which mutations in gene were detected. B, The number of patients with hotspot mutations. C, The number of patients with mutations in common drug target gene in all sample types. D, Genes whose mutations affected overall mutation load. E, Exemplary genes whose mutations did not affect overall mutation load. Mutation load was corrected by subtracting the mutations in the gene of interest from overall mutation load. The student *t* test was used to calculate P values between patients with (Mutated) or without (Wildtype) the mutation in the gene of interest

The frequencies of nonsilent mutations are shown in Figure 2A. The top eight mutated genes were TP53 (in 54.5% patients), NOTCH1 (36.4%), EGFR (32.7%), KDR (27.3%), PDGFRA (20.0%), ERBB2 (14.5%), JAK2 (14.5%), and ALK (12.7%) Furthermore, 45% (25/55) of patients were found to have known hotspot mutations defined by the COSMIC database (Figure 2B).

Mutations (n = 31) in actionable drug target genes were recurrently detected (Figure 2C), among which nine patients had EGFR p.G719A mutations, six patients had EGFR p.R776H mutations, and five patients possessed EGFR p.I646L. The Plasma Mutation Detector software not only detects mutations in PES, PEC, plasma, or tumor tissue samples, but it can also detect somatic mutations carried by normal tissues, the WBCs in this study (see Material and Methods). When applying Plasma Mutation Detector software to the WBC samples of 16 patients, we found four somatic mutations in three patients, two in TP53 gene and two in JAK3 gene (Table S6). However, these mutations were confined to WBC and were not found in other samples types used in this study. As previously reported,^33^ these mutations probably arose during clonal hematopoiesis.

Patients of the same cancer type usually carry a different number of mutations,^14^ and such variabilities (mutational load) have been shown to be closely associated with the efficacy of immunotherapy.^34^ Mutational load was calculated by dividing the mutation number of each patient by the target regions length (179 950 bp), and then normalizing this value to one million base pairs (mb). Mutation load ranged from 5.8 to 132 mutations/mb, with the mean value being 47 mutations/mb (Figure S4). The standard deviation reached 34.7 mutations/mb, almost 0.74 times that of the mean value (Figure S4). To examine whether patient mutation load varied with the presence of alterations in a specific gene, we divided patients into two groups according to the presence or absence of alterations in a specific gene (Figure 2D). As can be observed, the presence of alterations in the gene MET, NOTCH1, KDR, KRAS, APC, or BRAF, had a significant impact on total mutational load in the given patient (*P* < .05) (Figure 2D). This association was not observed for the gene TP53 or EGFR (Figure 2E). The patients (n = 5) with KRAS mutations had 61 mutations/mb, much higher than the patients (n = 47) without KRAS mutations (45 mutations/mb, *P* = .0042). Even more significant differences were observed with the KDR gene, which presented 68 mutations/mb in the patients with KDR mutations vs 34 mutations/mb in the patients without KDR mutations (*P* = 2.0 × 10^−4^). Pathway analysis indicates that these six genes are related to the endothelial cell chemotaxis and regulation of cell death pathways.

### 3.3 Comparison of OEC, tumor, pleural and plasma mutations

**Figure 3:**
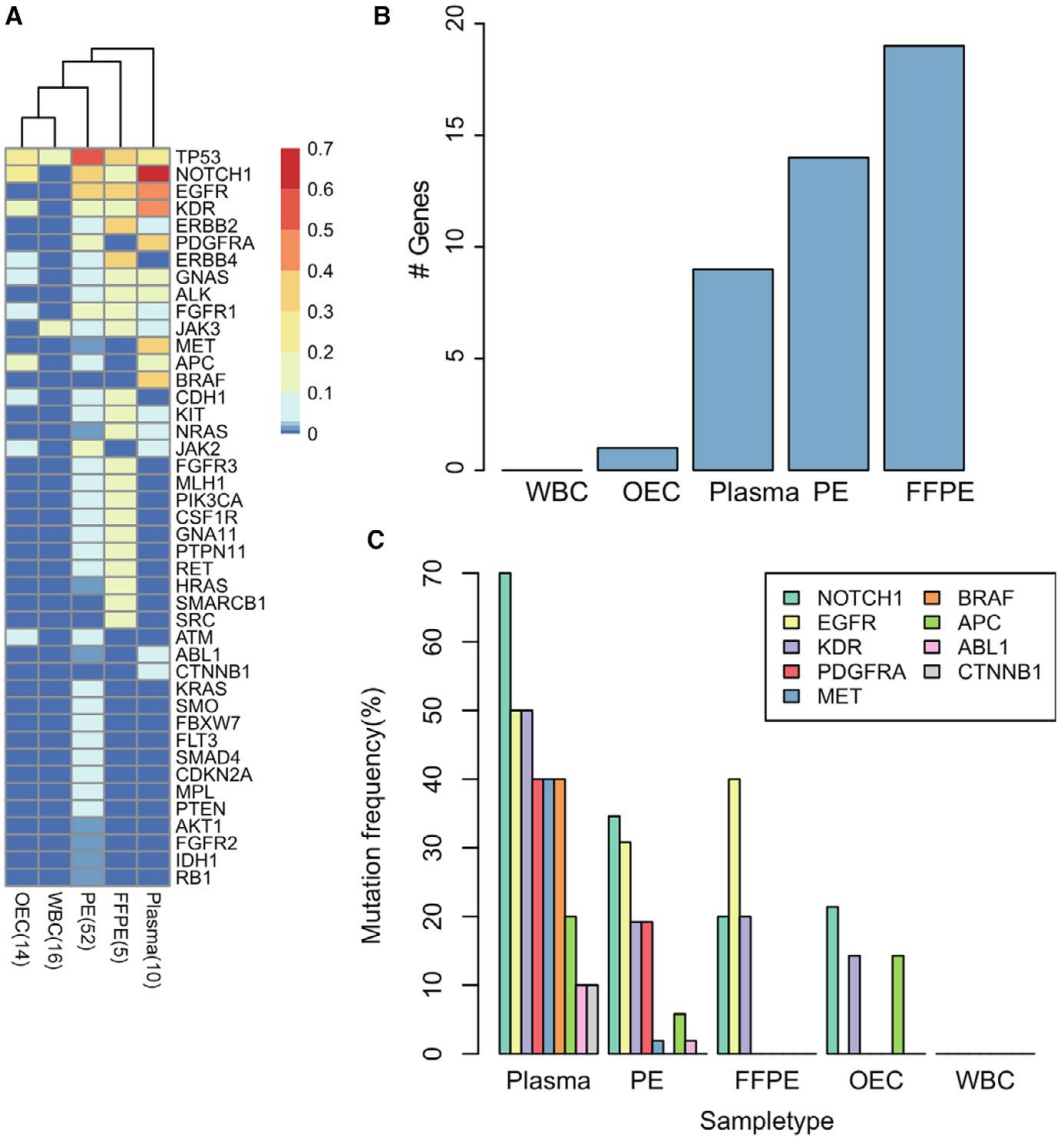
Heat map and Mutational frequency. A, Heat map of mutation frequencies for all genes that were mutated in at least one of the five sampling types. Mutation frequency refers to the percentage of mutated samples in each sample type. PE refers to the combination of PES and PEC. The FFPE (lymph node) and FFPE (tissue) of the same patient were merged into one. B, The number of genes that had the highest mutation frequency in a sample type. C, The mutation frequencies of genes that were most altered in plasma

Samples of different sources may reveal cancer mutations in different ways. PE is closer to the lung and thus may reflect the mutations of primary tumor and proximal metastasis. The plasma may contain gene mutations of primary tumor, proximal metastasis, and distant metastasis, due to the whole body circulation of peripheral blood. Herein, we analyzed the frequency of mutations within the 48 cancer gene panels in each sample type (Figure 3A). Although some level of mutational sharing was found among the samples, each sample source presented a high number of unshared mutations (Figure S5A‐D), suggesting the unique value of each sample type in revealing cancer genome alterations. OECs were sampled as a noncancerous source in 14 patients, with somatic mutations being found in seven patients. The most frequently altered gene in OEC was TP53 (Figure 3A). Interestingly, although still anecdotal due to low patient numbers, it was observed that in patients where tobacco consuming information was available, four of the five patients with OEC mutations were smokers, while only one of the five patients without OEC mutations smoked (Table S7). In the heat map (Figure 3A), the non‐cancerous samples of OEC and WBC clustered together and exhibited the lowest mutation frequency (Figure 3A). A majority of the genes (n = 19) showed the highest mutation frequency in tumor tissue, followed by PE (n = 14), and plasma (n = 9) (Figure 3B), which was consistent with the observation that tumor tissue had the highest number of mutations per patient (Figure S6). However, some genes, including NOTCH1, EGFR, KDR, MET and BRAF, displayed distinct behaviors; they reached their zenith of mutational frequency in plasma (Figure 3A,C). Especially NOTCH1, where 70% plasma samples exhibited at least one mutation, highlighting the unique values of plasma in detecting cancer mutations. This frequency is much higher than the frequency of 20% observed in our tumor samples and the 8% reported in the Cancer Genome Atlas.^16^ However, the NOTCH1 mutation frequency reported here is consistent with other cancer cohort studies, in terms of both lower mutation frequency in tumor tissues^14^, ^16^ and higher mutation frequency in cfDNAs.^21^, ^35^

**Figure 4:**
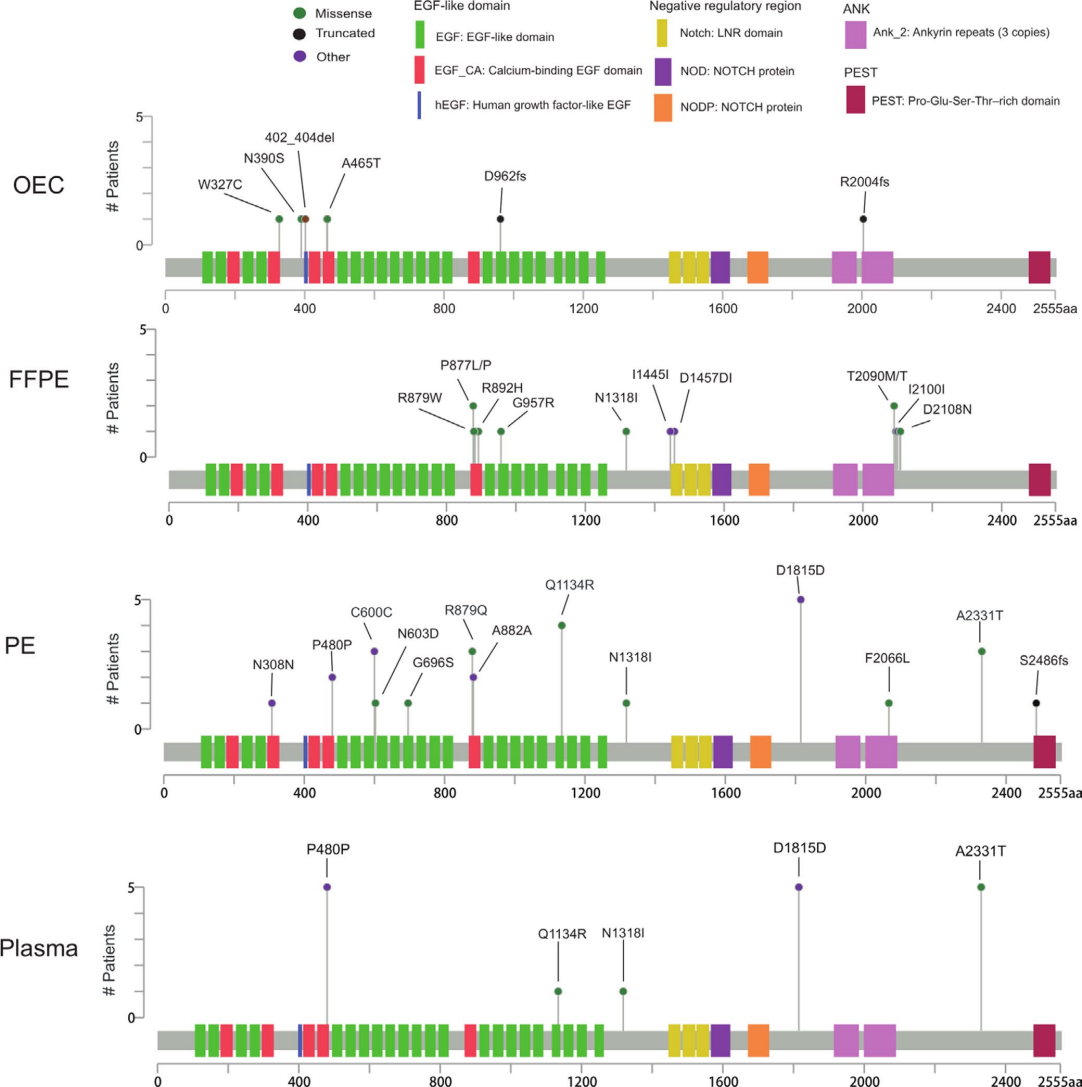
Schematic representation of NOTCH1 mutations among different sources of sample. Functional domains are marked by blocks of differing color along the NOTCH1 protein. Mutation types of missense (green), truncated (blue), and other (purple) are shown by lollipop symbols. The label, P877L/P, denotes two distinct mutations were identified at the 877 amino acid, the missense mutation from Proline (P) to Leucine (L) and the synonymous mutation from Proline to Proline. PE refers to the combination of PES and PEC

We conducted further analysis on NOTCH1 mutations across the different sample types (Figure 4 and Table S8). In total, 68 mutations were detected among 23 patients in PE (n = 28), plasma (n = 17), FFPE (n = 16), and OEC (n = 7). In FFPE and OEC, mutation distribution was stochastic; no one mutation occurred in more than one patient (Figure 4 and Table S8). In contrast, the tendency of clustering was obvious in PE and plasma. In PE, five mutations occurred in more than two patients. Mutations of plasma basically focused on three sites, NOTCH1 P480P, D1815D, and A2331T (Figure 4). Interestingly, each of the five mutations in plasma were also mutated in PE, but were not mutated in FFPE or OEC, indicating the close origination of these two fluid‐derived DNAs (Figure 4 and Table S8). Some mutations occurred in the important functional domains of NOTCH1, such as Calcium‐binding EGF domain, EGF‐like domain, LNR domain, Ankyrin repeats, and PEST domain (Figure 4), and thus may impair the NOTCH1 activity.

### 3.4 Copy number variants detected in the sediment of pleural effusions (PEC)

**Figure 5:**
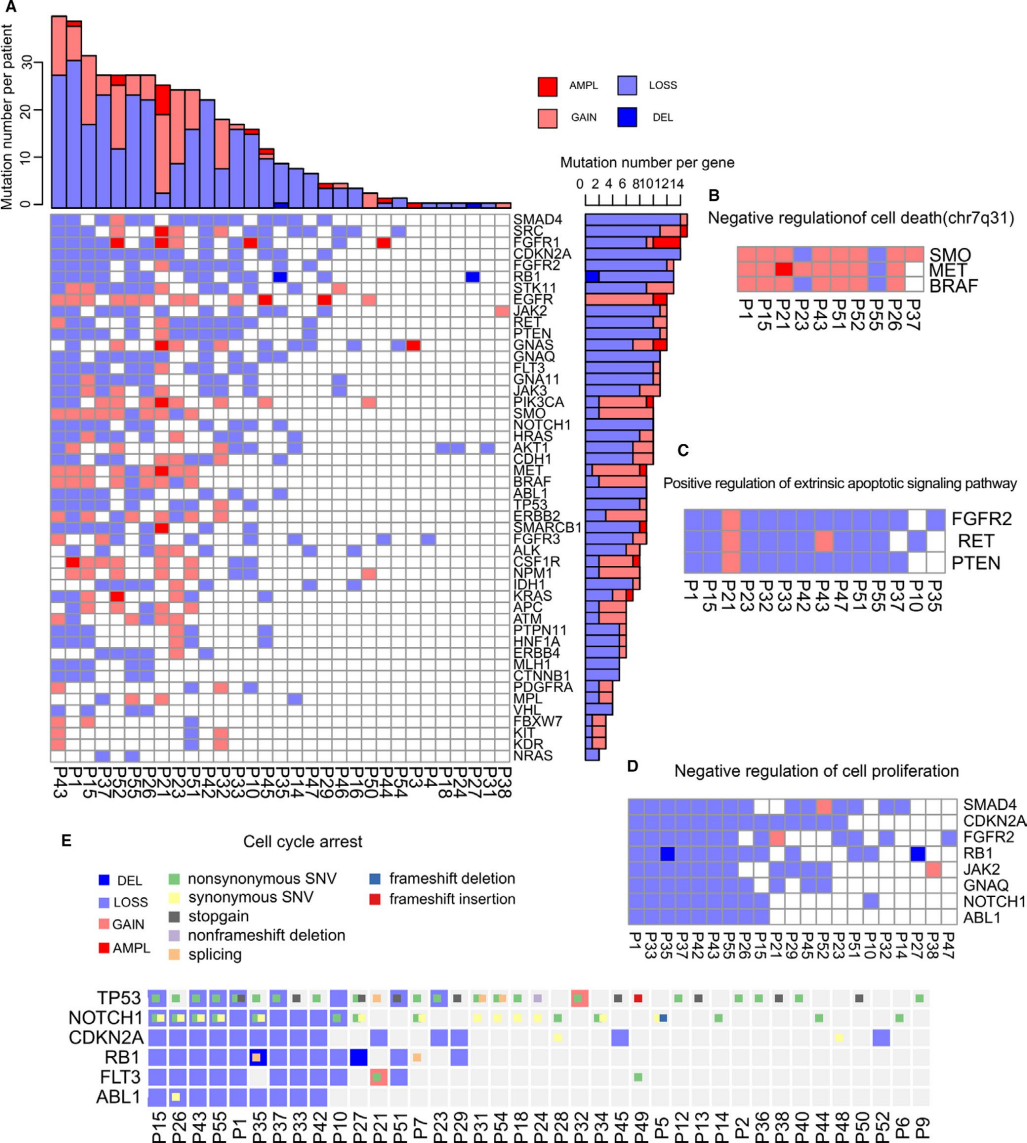
Landscape of copy number variants and associated biological pathways. A, Landscape of copy number variants in PEC samples. Copy number variants are shown for AMPL (dark red), Gain (light red), LOSS (light blue), and DEL (dark blue) across patients by columns and genes by rows. The top and right barplots show the number of CNVs in one patient or the number of patients who have CNVs in one gene and were sorted decreasingly. AMPL (amplification) means copy number is greater than or equal to 4, GAIN means copy number is between 2 and 4, loss means copy number is between 0 and 2 and DEL (deletion) means copy number is equal to 0. B‐D, Frequent and co‐concurrent copy number variants. The biological pathways shown in titles were inferred using GSEA. E, Concurrent analysis by combining mutations and CNVs. CNVs are shown as large color blocks in each grid, upon which gene mutations are overlaid as small color blocks

Gene function can be impaired by mutations and/or copy number variants (CNVs), both of which have been recurrently reported in lung cancer patients.^15^, ^36^, ^37^, ^38^ Although gene mutations have been extensively studied in target sequencing of various body fluids, CNVs have seldom been addressed.^39^ This may be due to body fluid‐derived cfDNA being inherently fragmented and its abundance affected by multiple factors, such as apoptosis, histone occupancy, as well as gene gain/loss.^24^ CNVs called from cfDNA‐based sequencing data are unstable and not biological relevant, which was the case when we tried to interpret cfDNA‐derived CNVs in this study (data not shown). In contrast, we obtained interesting findings in analyzing the CNVs from the sediment fraction of the PE samples. We speculated that PE cells contain intact genomes and thus the sequencing depth should more faithfully reflect copy number gain or loss of the tumor cells. In total, we found copy number variants in 66% (31/47) of PEC samples (Figure 5A). Among these 31 patients, 17 patients had copy number variants in drug target genes (Figure S7A). The top twelve genes with copy number alterations were SMAD4 (in 32% patients), SRC (32%), FGFR1 (30%), CDKN2A (30%), FGFR2 (28%), RB1 (28%), STK11 (28%), EGFR (26%), JAK2 (26%), RET (26%), PTEN (26%), and GNAS (26%) (Figure 5A). Copy number loss was much more prevalent than copy number gain (Figure 5A). The two “gain” CNV types, represented in Figure 5 as AMPL (amplified) and GAIN were identified in 17% and 34% patients respectively, while the two “loss” CNV types, LOSS and DEL (deleted), were identified in 57% and 4.3% patients respectively (Figure 5A). However, some genes, such as NPM1, EGFR, MET, SMO, BRAF, PI3KCA, CSF1R and ERBB2, principally exhibited copy number gain (Figure 5A). Of note was the EGFR gene which exclusively exhibited copy number gains (Figure 5A). A comparison with the Tumor Suppressor Gene Database (TSGene 2.0) indicated that these eight genes are known oncogenes^40^ (Figure S7B), and thus in our patient sample there is a significant enrichment of copy number gain in oncogenes (*P* = .0426, Fisher exact test). Highly amplified alleles (copy number greater than or equal to 4^41^) and represented as AMPL in Figure 5A, were rare in our lung cancer patients (3%). Eight genes, EGFR, FGFR1, GNAS, PI3KCA, MET, SMARCB1, KRAS and CSF1R, were identified as AMPL in at least one patient (Figure 5A). Pathway analysis using the Gene Set Enrichment Analysis (GSEA) from the Broad Institute^42^ indicates that these genes are related to protein tyrosine kinase activities or epithelium development (Figure S7C). In contrast to the overrepresentation of copy number gain in oncogenes, copy number loss was highly enriched in tumor suppressor genes (TSG) (Figure S7B). Among the 12 TSGs that exhibited copy number alterations in this study, eleven (91.7%) were copy number loss (Figure S7). Interestingly, CNV of some genes displayed a tendency of co‐occurrence (Figure 5B). The three genes involved in “negative regulation of cell death,” MET, SMO, and BRAF, concurrently exhibited the gain of copy number in seven patients (Figure 5B). In contrast, the three genes identified by GSEA to be involved in “Positive regulation of extrinsic apoptotic signaling pathway,” FGFR2, RET, and PTEN, simultaneously exhibited the loss of copy number in seven patients (Figure 5C). The simultaneous loss of these genes may increase the resistance of tumor cells to apoptosis. We also observed the consistent presence of copy number alterations in eight genes involved in “negative regulation of cell proliferation” (Figure 5D).

**Figure 6:**
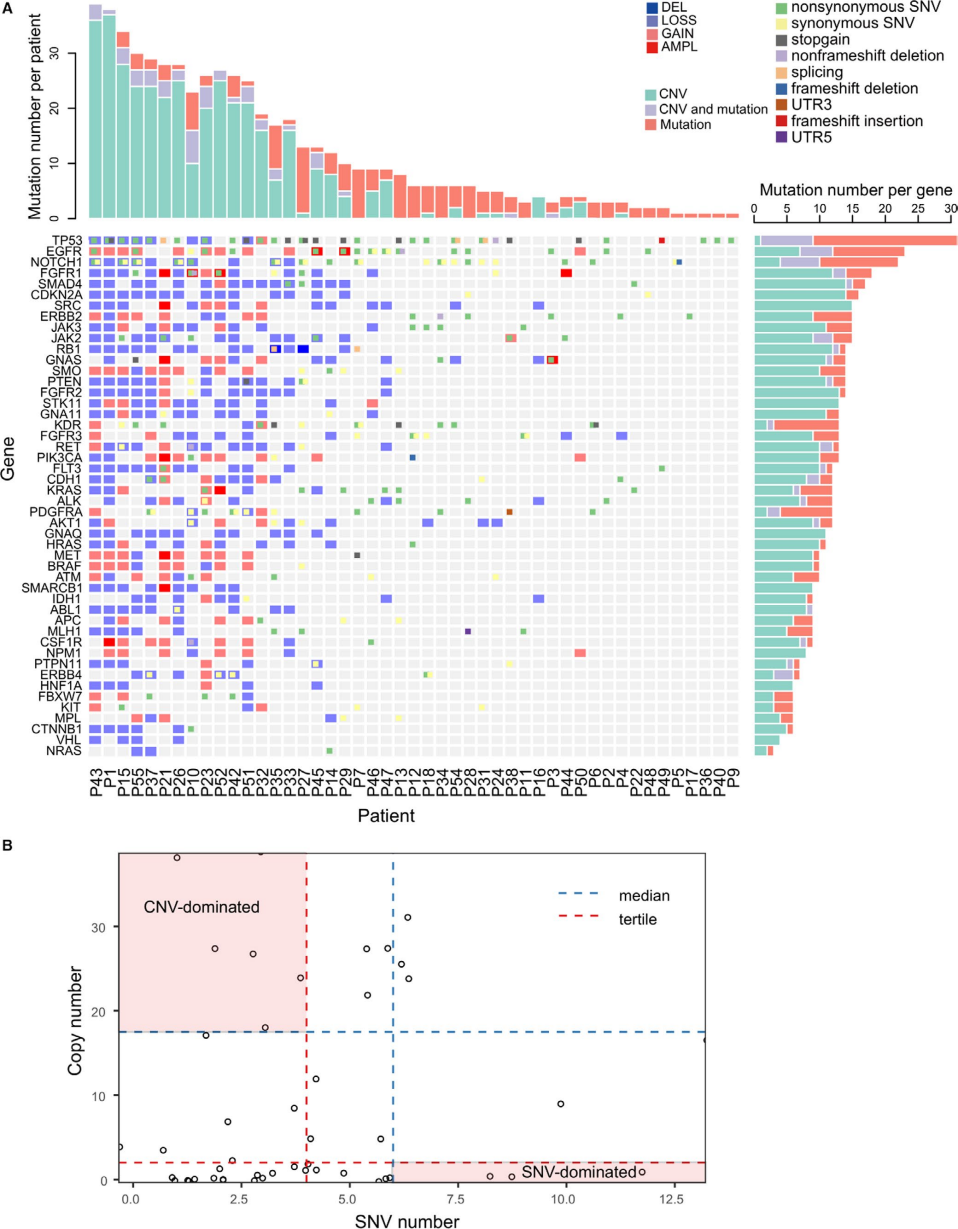
Scatter plots and landscape of copy number variants. A, Landscape of the concurrence of copy number variants and gene mutations. CNVs are shown as large color blocks in each grid, upon which gene mutations are overlaid as small color blocks. The right barplot shows the number of patients with only CNVs (green), mutations (red), or both CNVs and mutations (gray) in one gene. The top barplot shows the number of genes with only CNVs (green), mutations (red), or both CNVs and mutations (gray) in one patient. B, Scatter plot between SNV number and CNV number of patients. Patients were divided into CNV‐dominated, SNV‐dominated, and CNV and SNV codominated according to the relative amount of CNV and SNV in one patient

By combining CNVs and mutations, we identified a distinct gene set that potentially cooperates to protect tumor cells from cell cycle arrest (Figure 5E). Copy number loss of TP53, NOTCH1, CDKN2A, RB1, FLT3, and ABL1 was identified in 17%, 21%, 30%, 28%, 21%, and 19% patients respectively. Simultaneously, activity‐altering mutations, ie all the mutations except synonymous SNV (Figure 5E), were identified in TP53 (64%), NOTCH1 (38%), CDKN2A (4%), RB1 (4%), FLT (34%), and ABL1 (2%) of patients. Except for CDKN2A, all the other five genes exhibited some levels of coexistence of CNV and mutations, especially TP53 and NOTCH1, for which coexistence was identified in 17% and 11% patients, respectively (Figure 5E). We then thoroughly examined the coexistence of CNV and mutation for 48 genes in 47 patients (Figure 6). CNV aberration dominated in genes such as SRC and STK11, while mutational aberration dominated in KDR and PDGFRA. Coexistence of CNV and mutations were identified for most genes, most commonly for TP53, EGFR, and NOTCH1 (Figure 6A). Based on the relative number of CNVs or mutations, the patients can be divided into the three groups of CNV‐dominated, SNV‐dominated, or codominated (Figure 6B). These altogether indicate that copy number aberrations could be an important component of lung cancer progression, alone or together with SNVs.

## 4 DISCUSSION

Lung cancer has one of the highest levels of morbidity and mortality among malignancies, being responsible for approximately 1.38 million deaths worldwide every year.^43^ Targeted deep sequencing is a low cost and highly sensitive method for mutation detection. Gene mutation assessment in tumor biopsies is restrictive in accurately reflecting the genome of cancer cells that have escaped the primary tumor, which are often the population of cancer cells to which the drug therapy is directed. Furthermore, tumor biopsies are invasive, may have non‐ideal tissue locations and may lack adequate tissue acquisition.^44^, ^45^ Liquid biopsy, specifically cfDNA, has been proposed as a promising way to improve cancer diagnosis and prognosis.^46^

Our results are in general concordance with other deep sequencing studies in lung cancer patients, particularly within the Asiatic population.^25^ In total, in our study 153 samples were taken from 55 patients and analyzed on a 48 tumor gene panel. The presence of an FFPE, OECs, metastasis and blood cfDNA for every patient would have greatly enriched our analysis. However, our approach is still valid as the cancer cell genome is known to vary with the location of the tumor (eg primary tumor or metastasis) and even within the same tumor (ie multiply genome subsets with the primary tumor). Thus, mutation analysis from multiple sampling sources between patients still provides a valid picture of the genome alterations that occur between sampling locations.

Genomic alterations within our patients show consistency with the Catalogue Of Somatic Mutations In Cancer (COSMIC)^14^ and the comprehensive molecular profiling of lung adenocarcinoma (TCGA).^16^ Our study showed that TP53 was altered in 54.5% of patients, followed by EGFR (32.7%) and KRAS (10.9), which is consistent with the TCGA results that showed that TP53 was most frequently mutated (46% patients), with KRAS (33%) and EGFR (14%) among the most recurrent genes.^16^ A major difference between this study and the TCGA is that we analyzed samples from multiple sources and thus presumably detected mutations in both primary and metastatic tumors. Our TP53 mutation frequency of 54.5% dropped to 40% when analyses were confined exclusively to primary tumor samples (FFPE), close to the frequency (TP53, 46%; KRAS 25%) in a Finnish study of 425 FFPE samples.^47^

Our study used patients of Asian ancestry and demonstrated a higher mutation frequency among patients in EGFR than in KRAS. Discordance has been repeatedly noticed in the mutation frequency of EGFR and KRAS in the Asian vs Caucasian population. In Caucasian patients the mutation frequency is 14% for EGFR and 33% for KRAS.^16^ However, in a cohort of 306 lung cancer patients of Chinese ethnicity, the most frequently mutated gene was EGFR (46.7% of patients) and KRAS had a mutation frequency of only 10.1%.^25^ This Asian patient‐specific mutation pattern was confirmed by other studies that investigated 335^48^ and 112^49^ Chinese lung cancer patients, in which EGFR and KRAS were mutated in 39% (335 patient study), 51.79% (112 Patient study), and 11% (335 patient study), 8.93% (112 patient study), respectively. Thus our results clearly confirm the observation from previous studies that EGFR mutations are more prevalent than KRAS in Chinese lung cancer patients.

Patients with high mutation loads, ie, the number of mutations per million bases (mb) in target regions, are more susceptible to immune checkpoint inhibitors (eg PD‐1 or PD‐L1‐mediated immunotherapy) than the patients with low mutation loads in lung and other cancers.^34^, ^50^, ^51^ Some mutations in mismatch repair genes, homologous recombination genes, or POLE have been associated with high mutation load.^30^, ^52^ As previously reported, we found that mutation load varied among lung cancer patients and we identified six genes that could significantly affect patient mutation load^34^ (Figure 2D‐E). Interestingly NOTCH1 was significantly associated with mutational burden, as was MET, KDR, BRAF, APC and KAS, yet no association was observed with TP53 or EGFR.

Somewhat unexpectedly, we observed genetic alterations in non‐cancerous OECs. However, the vast majority of our patients in this study were smokers. Previous studies have shown that smokers possess more micronucleated oral mucosa cells than nonsmokers^53^ and the prevalence of aneuploidly has also been reported in apparently normal oral mucosa of heavy smokers.^54^ Moreover, the mutations present in the oral epithelial cells were also observed in the cancer samples, indicating these mutations could be cancerous. This is similar to a skin cell study that found that skin cells can carry cancer‐causing mutations while maintaining the physiological appearance and functions of the epidermis.^55^ Thus, although preliminary, our observations suggest that early genetic alterations can be detected in the oral mucosa of smokers and may in the future help screen smokers at risk for tobacco‐related cancers.

The discrepancy of NOTCH1 mutations in primary tumor and plasma has been previously reported.^16^, ^21^, ^35^ In the literature, the mutational frequency of NOTCH1 is reported to be 8% in the primary tumor^16^; however in a survey using cfDNA obtained from blood draws of small cell lung cancer patients, inactivating mutations in the NOTCH family of genes were observed in 52% of the patients.^35^ In another cfDNA study where NOTCH1 was analyzed, the authors reported that this gene was mutated with a frequency of 52.9%.^19^ In accordance, we observed a high NOTCH1 mutation in plasma (70%) and a low mutation in tumor tissue (20%) (Figure 3A). A further meta‐analysis of NSCLC patients indicated that elevated expression of NOTCH1 was associated with greater lymph node metastasis and higher TNM stage. Moreover, patients with NOTCH1 overexpression showed significantly poorer overall survival.^56^ From a clinical perspective, if only the primary tumor was biopsied, the presence of NOTCH1 mutations may be overlooked and therefore these subset of patients may not be considered as a target for precision medicine.

Targeting the NOTCH signaling pathway may benefit a subpopulation of NSCLC patients with NOTCH1 mutations. NOTCH1 is a receptor for the ligands Jagged1, Jagged2 and Delta1 that regulate cell‐fate determination.^57^ Upon ligand binding and activation, the notch intracellular domain is released and forms a transcriptional activator complex that subsequently activates genes of the enhancer of split locus to regulate cellular differentiation and development.^58^ As a well‐studied pathway it is not surprising that there already exists a therapy designed to inhibit this process. The humanized antibody brontictuzumab (OMP‐52M51) is designed to block the NOTCH1 receptor and has been shown in preclinical models to inhibit cancer stem cell growth and angiogenesis.^59^ Unfortunately, a combination of brontictuzumab together with chemotherapy was not tolerable in an initial trial in colon cancer patients.^60^ However, its safety and preliminary efficacy were encouragingly reported from a phase I study that administered this antibody intravenously to patients with hematologic malignancies.^61^ This, together with other methods to inhibit this signaling pathway, may find clinical use in patients who have been identified to carry alterations in the NOTCH1 gene by either mutations or CNVs. Depending on whether the drug target is the overall cancer burden or the remaining metastasis after primary tumor removal, we feel that mutations identified from multiple sampling sources will provide an accurate picture of the relevant cancer genome alterations and thus will deliver more tailored treatment regimens.

## 5 CONCLUSIONS

Our results confirm previous studies showing that EGFR mutations are more prevalent than KRAS in Chinese lung cancer patients, in contrast to previously reported Caucasian populations. We observed a high NOTCH1 mutation rate in plasma in comparison to tumor tissue, which may reveal a role of NOTCH1 modifications in tumor metastasis and highlight the clinical advantage of the plasma in detecting mutation biopsy.

## CLINICAL PRACTICE POINTS

Most of the lung cancer genome studies were performed on samples of tumor tissue (FFPE or fresh frozen tissue). However, the genomic spectrum varies across lesion sites for most solid tumors; thus, a single specimen typically underestimates the number of mutations, and target therapy may be challenged by intratumoral heterogeneity. In this study, we compared somatic mutations from six sources of samples including PES, PEC, plasma, tumor tissues, WBC, and OEC using deep sequencing of targeted genome composed of the full length of 48 cancer‐related genes. Copy number variants were also identified in PEC samples. This study brings attention to demonstrate the unique values of each sample type for the future personalization of lung cancer, treatment and will benefit the clinical design and implementation of liquid biopsies.

## Data Availability

The datasets used and analysed during the current study are available from the corresponding author on reasonable request.
