# Supplementary figures and images for: Targeted deep sequencing from multiple sources demonstrates increased NOTCH1 alterations in lung cancer patient plasma
Source: Cancer Med. 2019 Aug 1;8(12):5673–86. doi: 10.1002/cam4.2458 (PMC6745866; doi:10.1002/cam4.2458)

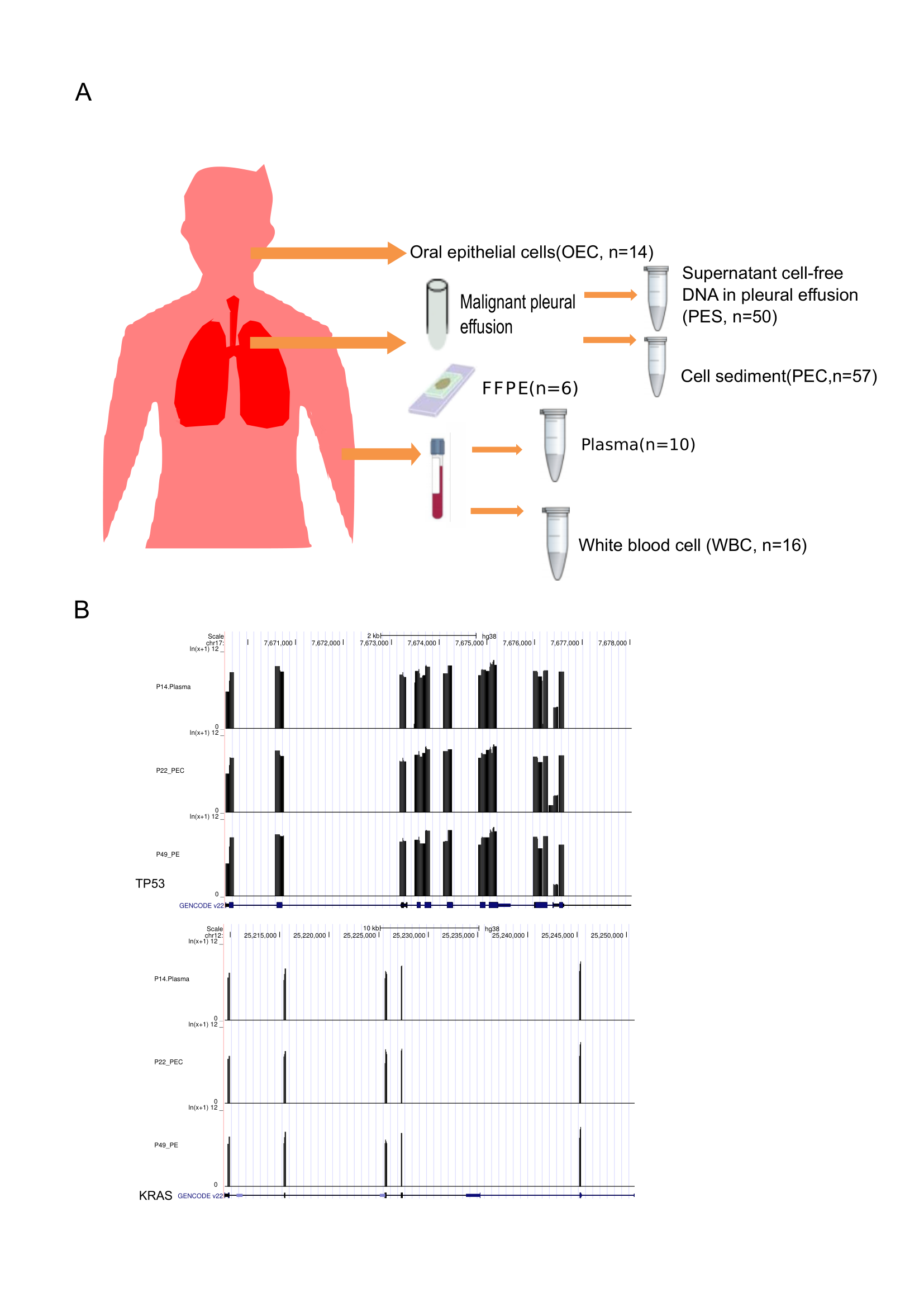

Supplement: Supplementary file 1 [file CAM4-8-5673-s001.tiff]

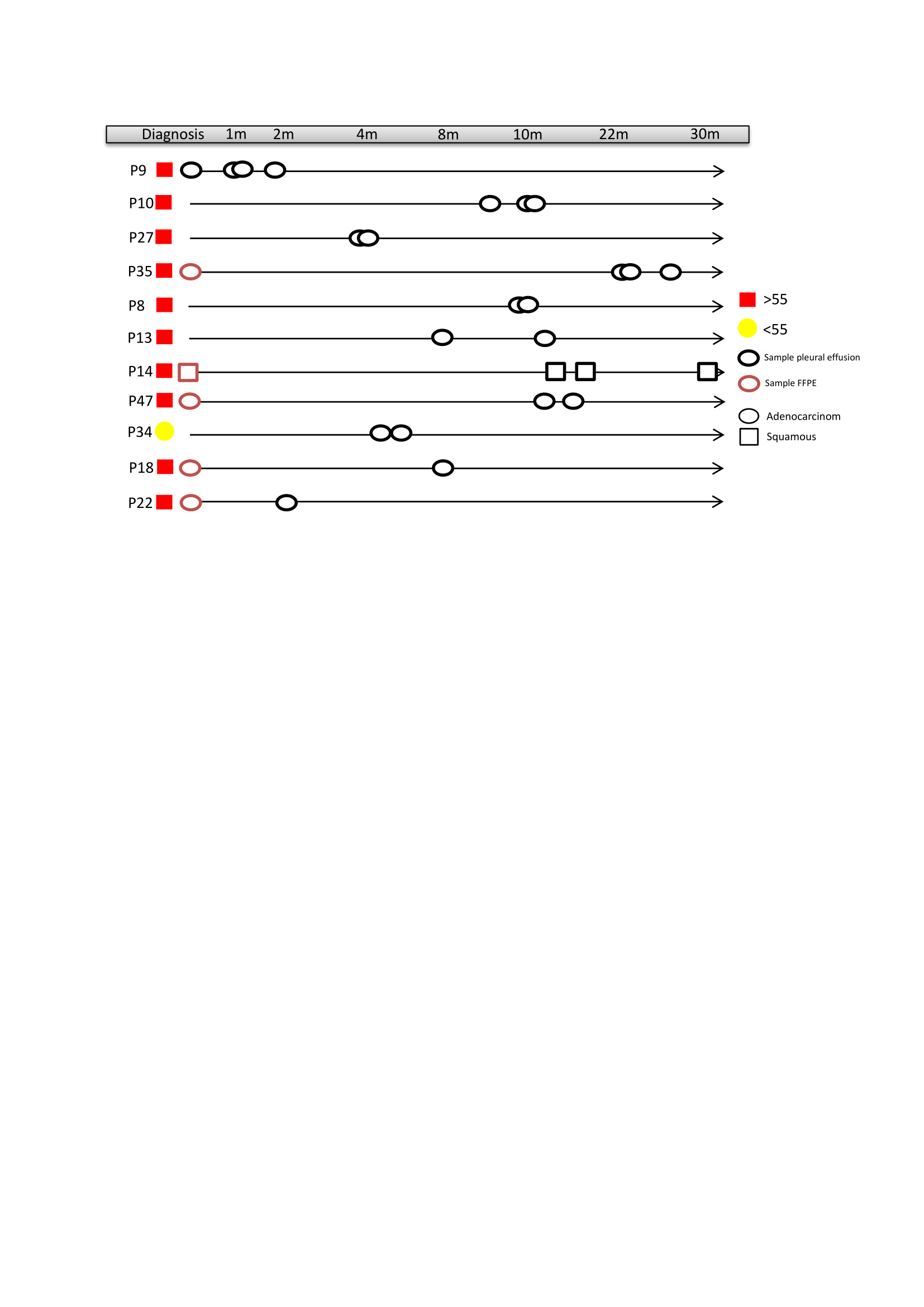

Supplement: Supplementary file 2 [file CAM4-8-5673-s002.tiff]

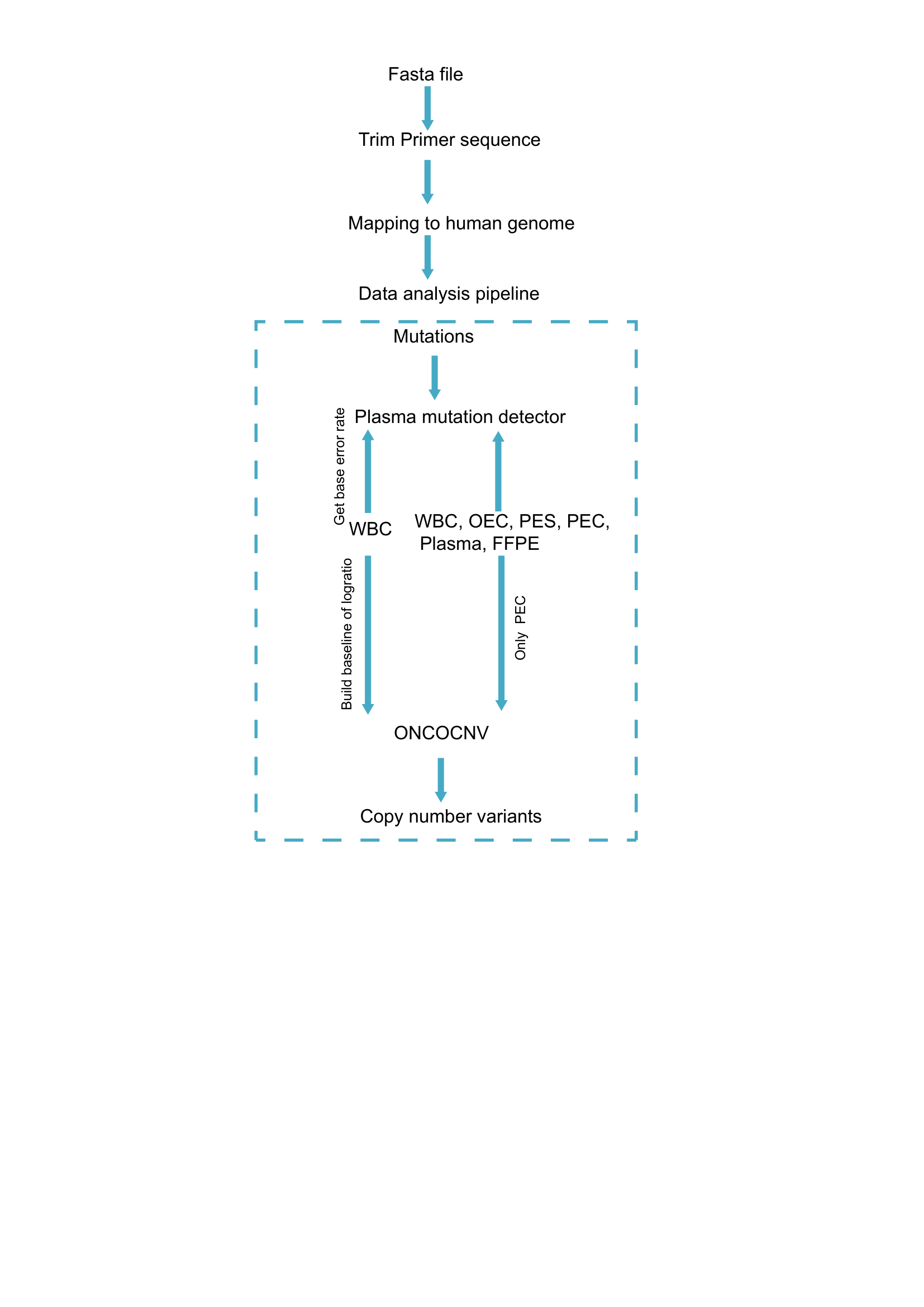

Supplement: Supplementary file 3 [file CAM4-8-5673-s003.tiff]

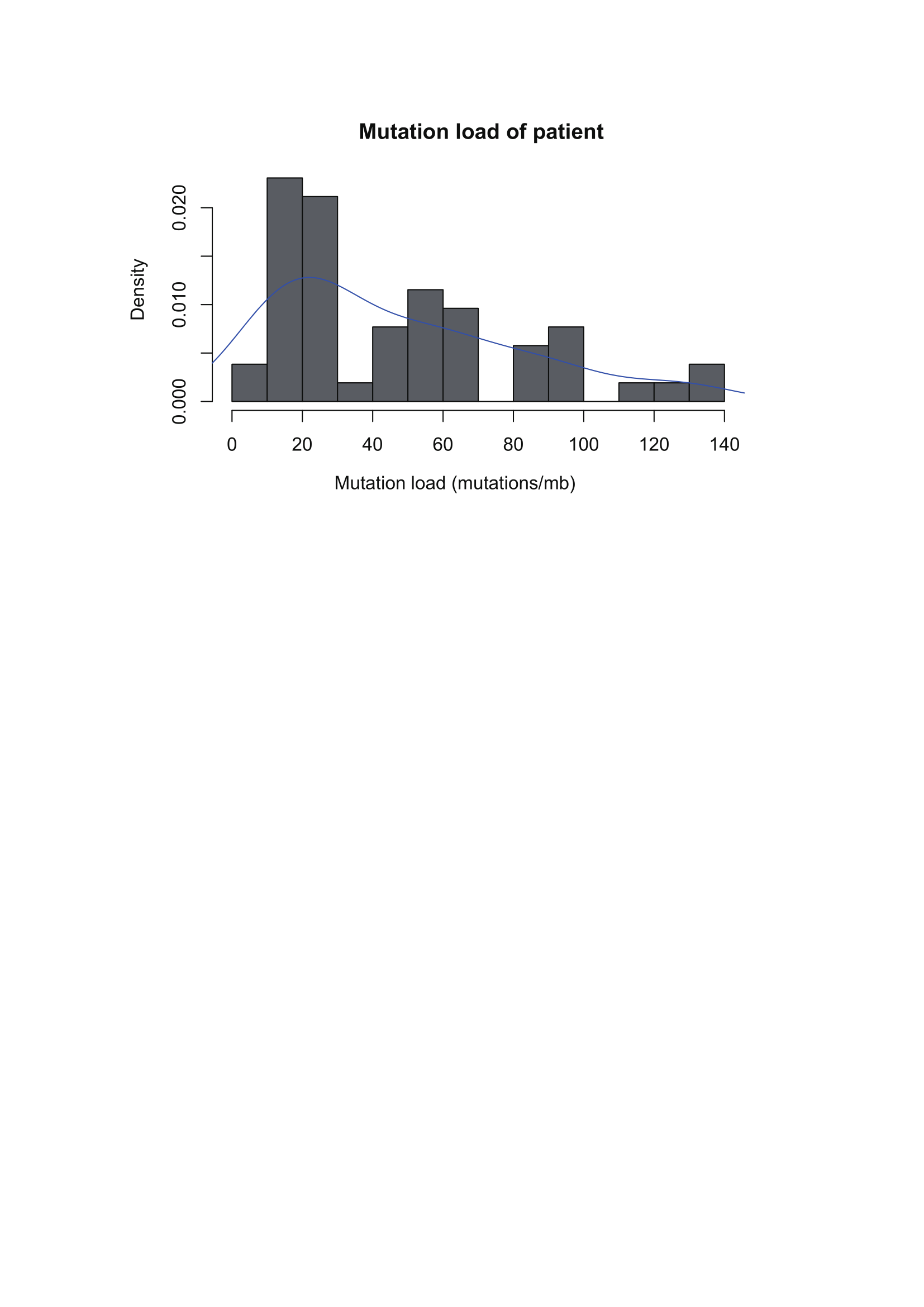

Supplement: Supplementary file 4 [file CAM4-8-5673-s004.tiff]

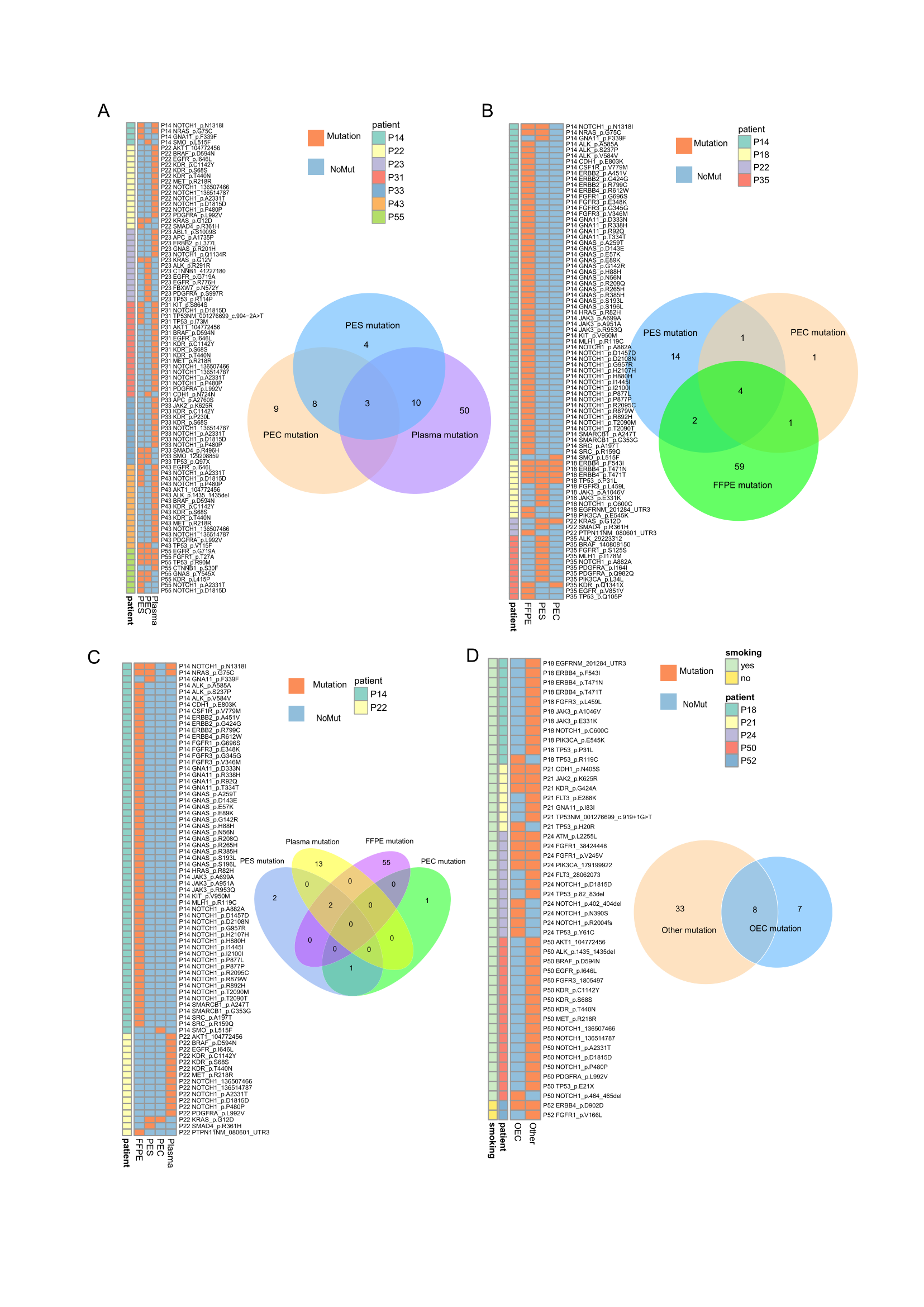

Supplement: Supplementary file 5 [file CAM4-8-5673-s005.tiff]

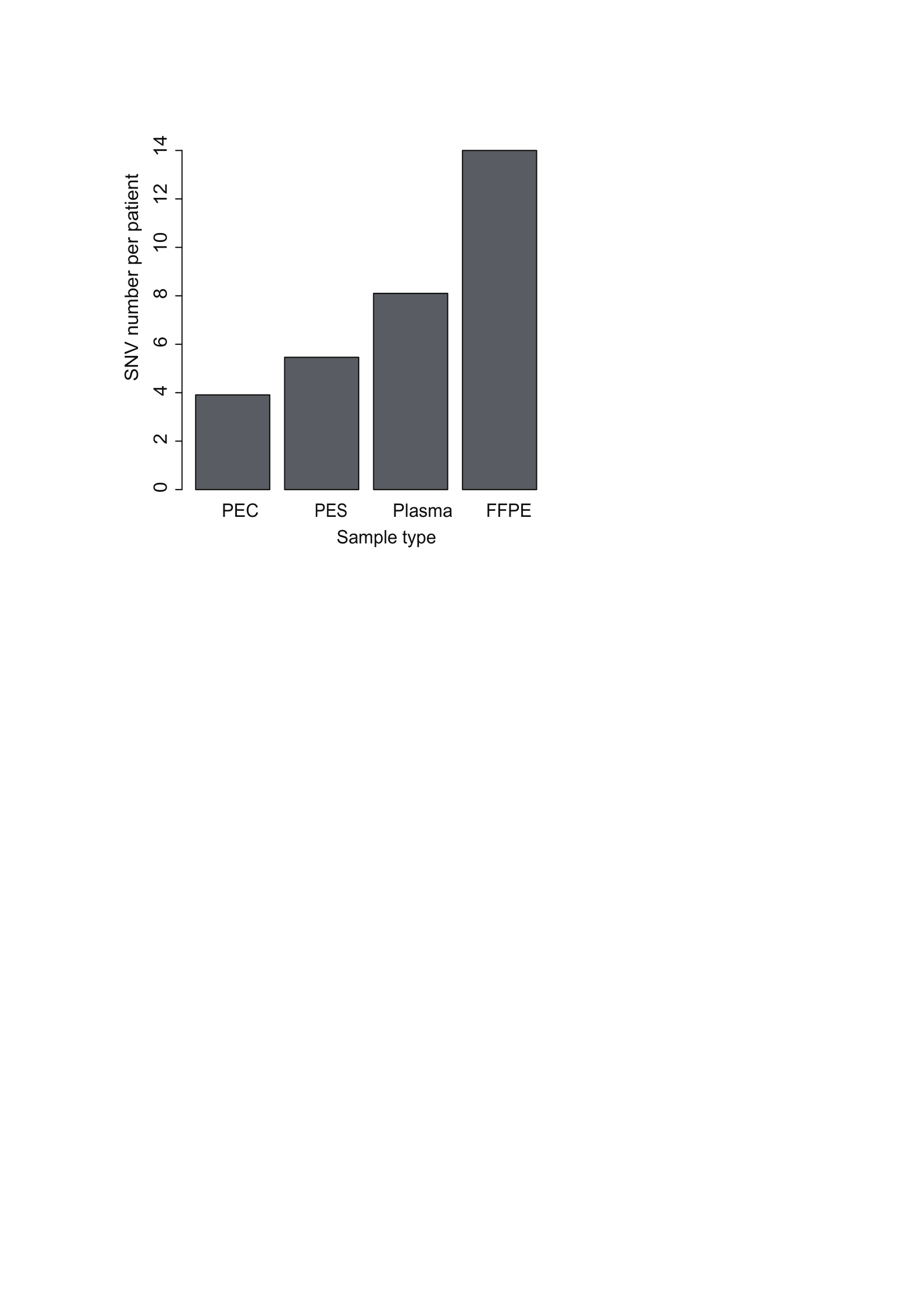

Supplement: Supplementary file 6 [file CAM4-8-5673-s006.tiff]

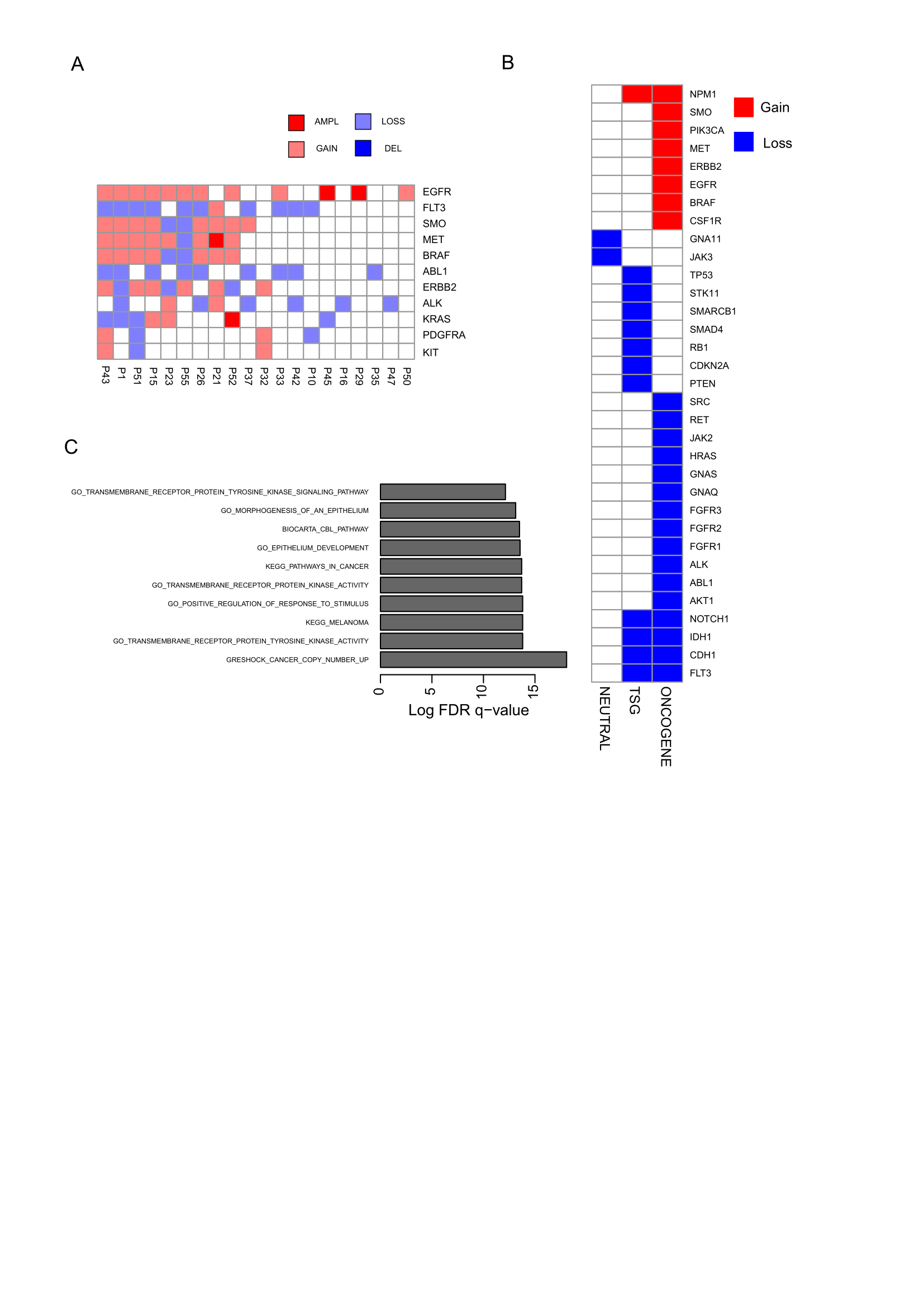

Supplement: Supplementary file 7 [file CAM4-8-5673-s007.tiff]
